# Organizational Justice, Perceived Organizational Support, and Computer Self-Efficacy in Knowledge Sharing Among Healthcare Workers: The Mediating Role of Organizational Citizenship Behavior

**DOI:** 10.3390/healthcare14040463

**Published:** 2026-02-12

**Authors:** Chen-Wei Yang, Sian-Hao Huang, Yu-Hua Yan

**Affiliations:** 1 Department of Health-Business Administration, Fooyin University, No. 151, Jinxue Rode, Daliao District, Kaohsiung City 831301, Taiwan; xx709@fy.edu.tw (C.-W.Y.); sianhaohuang@gmail.com (S.-H.H.); 2 Superintendent Office, Tainan Municipal Hospital (Managed by Show Chwan Medical Care Corporation), No. 670, Chongde Rode, East District, Tainan City 701, Taiwan

**Keywords:** organizational justice, perceived organizational support, organizational citizenship behavior, knowledge sharing, computer self-efficacy, healthcare organizations

## Abstract

**Background:** Knowledge sharing is essential for healthcare organizations to enhance service quality, organizational learning, and sustainable performance. However, the organizational and individual conditions that facilitate voluntary knowledge sharing among healthcare workers remain insufficiently explored. **Methods:** This cross-sectional study surveyed healthcare employees working in hospital organizations in Taiwan. A total of 355 valid questionnaires were analyzed using SPSS 22. Descriptive statistics, correlation analysis, hierarchical regression analysis, and mediation testing were conducted to examine the relationships among organizational justice, perceived organizational support, computer self-efficacy, organizational citizenship behavior (OCB), and knowledge sharing. **Results:** The results indicated that organizational justice, perceived organizational support, and computer self-efficacy were positively associated with organizational citizenship behavior. Organizational citizenship behavior, in turn, had a significant positive effect on knowledge sharing. Moreover, organizational citizenship behavior partially mediated the relationships between organizational justice and knowledge sharing, as well as between perceived organizational support and knowledge sharing. In contrast, the mediating effect of organizational citizenship behavior between computer self-efficacy and knowledge sharing was not supported. **Conclusions:** The findings highlight the pivotal role of organizational citizenship behavior in translating favorable organizational conditions into knowledge-sharing behaviors in healthcare settings. Creating fair and supportive organizational environments appears to be more critical than individual technical confidence alone in promoting sustainable knowledge sharing among healthcare workers.

## 1. Introduction

Healthcare organizations operate in highly complex and knowledge-intensive environments characterized by rapid technological advancement, increasing service demands, workforce shortages, and continuous policy and reimbursement reforms. In such contexts, effective knowledge sharing among healthcare workers has been widely recognized as a critical driver of service quality, organizational learning, and sustainable performance [1,2,3]. Clinical expertise, procedural know-how, and experiential knowledge embedded in healthcare professionals constitute valuable organizational assets that, when effectively shared, can enhance coordination, reduce errors, and improve patient outcomes.

From the knowledge-based view, organizational competitiveness depends not only on the possession of knowledge but also on the ability to integrate and disseminate knowledge across organizational members [2,4]. In healthcare settings, however, knowledge sharing is particularly challenging due to professional boundaries, hierarchical structures, time pressure, and uneven workload distribution [5]. These characteristics make voluntary and informal knowledge exchange essential yet difficult, highlighting the importance of understanding behavioral mechanisms that facilitate knowledge sharing among healthcare workers [6].

Prior research has identified organizational citizenship behavior (OCB) as a key mechanism that supports knowledge sharing. OCB refers to discretionary, extra-role behaviors that are not formally rewarded but contribute to organizational effectiveness, such as helping colleagues, sharing expertise, and voluntarily supporting organizational initiatives [7]. In healthcare organizations, where formal job descriptions cannot fully capture the complexity of daily work, OCB plays a crucial role in sustaining collaboration and informal knowledge exchange. Employees who demonstrate higher levels of OCB are more likely to engage in proactive knowledge sharing, thereby strengthening collective competence within healthcare teams [8,9,10].

Although the positive relationship between OCB and knowledge sharing has been well documented, relatively less attention has been paid to the organizational antecedents that foster OCB in healthcare contexts. Organizational justice and perceived organizational support are two important organizational conditions grounded in social exchange theory that shape employees’ discretionary behaviors [11,12]. Organizational justice reflects employees’ perceptions of fairness in decision-making processes, resource allocation, and interpersonal treatment [13]. Empirical studies have consistently shown that when employees perceive higher levels of organizational justice, they are more likely to reciprocate through positive attitudes and behaviors, including OCB [14,15,16].

Similarly, perceived organizational support captures the extent to which employees believe that their organization values their contributions and cares about their well-being [12]. Employees who perceive strong organizational support tend to develop a sense of obligation to help the organization achieve its goals, which in turn encourages extra-role behaviors and cooperative actions [17]. In healthcare environments characterized by high stress and emotional demands, perceived organizational support may play an especially important role in motivating healthcare workers to engage in citizenship behaviors that facilitate knowledge sharing [18]. Recent research also points out that leadership styles such as servant leadership can foster knowledge sharing by enhancing psychological safety and trust among employees, illustrating a behavioral pathway through which organizational context promotes voluntary knowledge exchange [19].

In addition to organizational factors, individual-level technological confidence may also influence knowledge-sharing behaviors. With the increasing digitalization of healthcare systems, computer self-efficacy—defined as an individual’s belief in their ability to use computer technologies effectively—has become increasingly relevant [20]. Individuals with higher computer self-efficacy are more confident in using information systems and digital platforms, which may lower technical barriers to knowledge sharing. However, empirical findings regarding the role of computer self-efficacy in promoting discretionary behaviors remain mixed, suggesting that technological confidence alone may not be sufficient to stimulate voluntary knowledge sharing in organizational settings [21,22,23].

Despite the growing body of literature on knowledge sharing and organizational behavior, several gaps remain. First, existing studies often examine organizational justice, perceived organizational support, or computer self-efficacy in isolation, rather than integrating these factors into a unified analytical framework. Second, empirical research focusing specifically on healthcare organizations remains limited, even though healthcare workers face unique professional and organizational pressures that may shape their behavioral responses. Third, the mediating role of organizational citizenship behavior in linking organizational and individual antecedents to knowledge sharing has not been sufficiently examined in healthcare contexts.

To address these gaps, this study develops and empirically tests an integrated model examining the relationships among organizational justice, perceived organizational support, computer self-efficacy, organizational citizenship behavior, and knowledge sharing within healthcare organizations. By positioning OCB as a mediating mechanism, this study aims to clarify how favorable organizational conditions and individual technological confidence translate into knowledge-sharing behaviors. Using survey data collected from healthcare employees in hospital settings, this research contributes to the literature by providing empirical evidence on the behavioral pathways that promote knowledge sharing in healthcare organizations.

## 2. Literature Review and Hypotheses

### 2.1. Knowledge Sharing in Healthcare Organizations

Knowledge sharing refers to employees’ activities of exchanging task-related information, know-how, and experience with colleagues to enhance collective performance and problem-solving capacity [9]. In knowledge-intensive settings such as hospitals, knowledge sharing supports clinical coordination, service improvement, and organizational learning, enabling organizations to transform individual expertise into organizational capability [1,2]. Recent research further emphasizes that effective knowledge sharing among healthcare professionals contributes to care quality, patient safety, and organizational adaptability in complex healthcare systems [3].

However, knowledge sharing is often discretionary rather than mandated; it is influenced by both the organizational context and employees’ willingness to go beyond formal role requirements [8,9]. Contemporary studies highlight that in healthcare organizations, informal and voluntary knowledge exchange remains essential but challenging due to professional boundaries, time constraints, and hierarchical structures [6]. Therefore, identifying behavioral and contextual drivers of knowledge sharing is essential for effective healthcare management.

### 2.2. Organizational Citizenship Behavior

Organizational citizenship behavior (OCB) describes discretionary behaviors that are not explicitly recognized by formal reward systems but contribute to organizational effectiveness [7]. In hospitals, OCB—such as voluntarily helping coworkers, sharing professional tips, and supporting team functioning—creates a cooperative climate and reduces coordination costs. Because knowledge sharing frequently requires time, effort, and interpersonal engagement, it often resembles a form of extra-role contribution. Accordingly, OCB can be conceptualized as a key behavioral mechanism that facilitates voluntary knowledge sharing in healthcare organizations.

### 2.3. Organizational Justice and Organizational Citizenship Behavior

Organizational justice captures employees’ perceptions of fairness in organizational procedures, outcomes, and interpersonal treatment [24]. Justice perceptions signal whether the organization allocates resources and makes decisions in a trustworthy and respectful manner. From a social exchange perspective, perceptions of fairness signal organizational trustworthiness and activate reciprocity norms [11], motivating employees to voluntarily contribute beyond formal job requirements.

A substantial body of empirical research has consistently linked organizational justice to organizational citizenship behavior, demonstrating that employees who perceive fairness are more willing to exert extra effort, cooperate with colleagues, and engage in helping behaviors [13,14,15]. Recent meta-analytic evidence further reinforces the robustness of the justice–OCB relationship across organizational contexts, underscoring justice as a key driver of citizenship behavior [16]. In hospital settings—where workload intensity and interprofessional collaboration are common—justice perceptions may be particularly salient for motivating citizenship behaviors.

**H1:**  *Organizational justice positively affects organizational citizenship behavior among hospital members.*

### 2.4. Perceived Organizational Support and Organizational Citizenship Behavior

Perceived organizational support (POS) refers to employees’ belief that the organization values their contributions and cares about their well-being [12]. POS strengthens employees’ sense of obligation and attachment to the organization, thereby encouraging prosocial and extra-role behaviors. Theoretically, POS operates through reciprocity norms: when employees feel supported, they are motivated to repay the organization through behaviors that benefit the workplace, including organizational citizenship behavior [11,12].

Recent empirical studies continue to confirm the positive relationship between perceived organizational support and organizational citizenship behavior, suggesting that supportive organizational environments play a crucial role in sustaining discretionary behaviors, particularly in high-demand service contexts such as healthcare [18]. Given the emotional labor and stress inherent in healthcare work, perceived organizational support may be especially influential in motivating hospital employees to engage in citizenship behaviors that facilitate cooperation and knowledge sharing.

**H2:**  *Perceived organizational support positively affects organizational citizenship behavior among hospital members*

### 2.5. Computer Self-Efficacy and Organizational Citizenship Behavior

Computer self-efficacy (CSE) is an individual’s belief in their capability to use computer technologies to accomplish tasks [20]. In healthcare organizations increasingly reliant on information systems, CSE may reduce technology-related anxiety and increase employees’ confidence in using digital tools for communication and collaboration [20,21]. As employees become more competent in handling digital workflows, they may be more willing to assist colleagues and contribute proactively, behaviors that are consistent with organizational citizenship behavior.

Prior research suggests that efficacy beliefs influence motivation, learning, and performance-related behaviors in technology-rich environments [21,25]. However, recent studies caution that technological competence alone does not automatically translate into collaborative or prosocial behaviors, indicating the importance of considering behavioral and organizational contexts alongside individual self-efficacy [23].

**H3:**  *Computer self-efficacy positively affects organizational citizenship behavior among hospital members.*

### 2.6. Organizational Citizenship Behavior and Knowledge Sharing

Organizational citizenship behavior plays a critical role in facilitating knowledge sharing by fostering cooperation, trust, and reciprocal relationships among employees. Because knowledge sharing frequently requires individuals to invest time and effort without immediate rewards, it closely aligns with the nature of extra-role behaviors [7]. Prior studies indicate that individuals who demonstrate higher levels of organizational citizenship behavior are more likely to engage in proactive knowledge exchange, thereby strengthening collective learning and team effectiveness [8,9].

More recent empirical evidence further confirms that organizational citizenship behavior serves as a key behavioral antecedent of knowledge sharing by promoting collaborative norms and interpersonal trust within organizations [10]. Accordingly, organizational citizenship behavior is expected to positively influence knowledge sharing among hospital employees.

**H4:**  *Organizational citizenship behavior positively affects knowledge sharing among hospital members.*

### 2.7. The Mediating Role of Organizational Citizenship Behavior

Although organizational justice, perceived organizational support, and computer self-efficacy may influence knowledge sharing, their effects are likely to be realized through discretionary behavioral mechanisms. Organizational citizenship behavior represents such a mechanism because it reflects employees’ willingness to voluntarily contribute to organizational functioning and coworker support [7]. Drawing on social exchange theory, employees who experience fairness and organizational support are more likely to engage in organizational citizenship behavior, which in turn fosters a cooperative climate and increases the likelihood of knowledge sharing [11,13,17].

Recent research further supports the mediating role of organizational citizenship behavior in translating organizational conditions into knowledge-sharing behaviors, particularly in knowledge-intensive and service-oriented contexts [10,18]. Similarly, higher computer self-efficacy may encourage proactive helping behaviors through increased confidence in technology-enabled collaboration, although its indirect effect on knowledge sharing remains subject to contextual factors. Accordingly, this study proposes organizational citizenship behavior as a mediator linking organizational and individual antecedents to knowledge sharing in healthcare organizations.

**H5-1:**  *Organizational citizenship behavior mediates the relationship between organizational justice and knowledge sharing.*

**H5-2:**  *Organizational citizenship behavior mediates the relationship between perceived organizational support and knowledge sharing.*

**H5-3:**  *Organizational citizenship behavior mediates the relationship between computer self-efficacy and knowledge sharing.*

## 3. Method

### 3.1. Study Design and Research Framework

This study employed a cross-sectional survey design to examine the relationships among organizational justice, perceived organizational support, computer self-efficacy, organizational citizenship behavior, and knowledge sharing within healthcare organizations. Based on the theoretical foundations of social exchange theory and the knowledge-based view, an integrated research framework was developed in which organizational justice, perceived organizational support, and computer self-efficacy were specified as antecedents of organizational citizenship behavior, which in turn was hypothesized to influence knowledge sharing.

### 3.2. Participants and Data Collection

The study population consisted of employees working in hospital organizations in Taiwan. A structured questionnaire survey was administered to healthcare employees across different departments. A total of 400 questionnaires were distributed, and 355 valid responses were retained after data screening, yielding an effective response rate of 88.75%. Questionnaires with excessive missing values or patterned responses were excluded to ensure data quality.

### 3.3. Measurement Instruments

All constructs were measured using previously validated scales adapted to the healthcare context. Responses were recorded on a five-point Likert scale ranging from 1 (“strongly disagree”) to 5 (“strongly agree”).

#### 3.3.1. Organizational Justice

Organizational justice was measured using the scale developed by [13], translated and adapted for the Taiwanese context. The scale captures three dimensions: distributive justice, procedural justice, and interactional justice. Higher scores indicate stronger perceptions of fairness within the organization.

#### 3.3.2. Perceived Organizational Support

Perceived organizational support was assessed using items adapted from [12], which measure employees’ perceptions of the extent to which the organization values their contributions and cares about their well-being.

#### 3.3.3. Computer Self-Efficacy

Computer self-efficacy was measured using the scale developed by [20]. This scale evaluates respondents’ confidence in their ability to use computer technologies to accomplish work-related tasks.

#### 3.3.4. Organizational Citizenship Behavior Scale

Organizational citizenship behavior was measured using a multidimensional scale adapted from prior research, including dimensions such as altruism, conscientiousness, sportsmanship, courtesy, and civic virtue [7,26].

#### 3.3.5. Knowledge Sharing

Knowledge-sharing behavior was assessed using a scale adapted from prior knowledge management studies, capturing respondents’ willingness to share information, experience, and expertise with colleagues [8,9].

### 3.4. Reliability and Validity

Internal consistency reliability was evaluated using Cronbach’s alpha coefficients. All constructs exceeded the commonly accepted threshold of 0.70, indicating satisfactory reliability. Content validity was ensured through expert review prior to survey administration.

Although confirmatory factor analysis (CFA) is commonly recommended for theory-driven measurement validation, exploratory factor analysis (EFA) was employed in this study for two reasons. First, several measurement scales were adapted and contextually modified for healthcare workers in Taiwan, necessitating an initial examination of the factor structure within this specific organizational and cultural context. Second, the primary analytical focus of this study was on regression-based mediation analysis rather than latent variable modeling.

Accordingly, construct validity was examined using exploratory factor analysis. The results supported the expected factor structure, with factor loadings exceeding recommended thresholds, indicating satisfactory convergent validity. Nevertheless, we acknowledge that EFA has limitations in testing discriminant validity compared to CFA. Therefore, future research is encouraged to apply confirmatory factor analysis or structural equation modeling to further validate the measurement model and strengthen construct validity.

### 3.5. Data Analysis

Data analysis was conducted using IBM SPSS Statistics (Version 22.0, IBM Corp., Armonk, NY, USA). Descriptive statistics were used to summarize participant characteristics. Pearson correlation analysis was performed to examine associations among variables. Hierarchical regression analysis was applied to test the direct effects proposed in the hypotheses. Mediation effects were tested using hierarchical regression analysis in combination with a bootstrapping approach to assess the significance of indirect effects, with organizational citizenship behavior specified as the mediating variable.

#### Regression Assumption Testing

Prior to conducting hierarchical regression analyses, a series of diagnostic tests was performed to examine whether the assumptions of linear regression were adequately met. The normality of residuals was assessed through visual inspection of standardized residual histograms and normal probability (P–P) plots, which indicated approximately normal distributions. Homoscedasticity was evaluated using scatterplots of standardized residuals against predicted values, revealing no evident patterns or funnel effects.

Potential outliers were examined using standardized residuals and Cook’s distance statistics, and no influential observations exceeding commonly accepted thresholds were identified. The independence of residuals was verified using the Durbin–Watson statistic, with values close to 2.0 indicating no serious autocorrelation. Multicollinearity was assessed by examining variance inflation factors (VIFs) and tolerance values, all of which fell within acceptable ranges. These diagnostic results support the appropriateness of applying linear regression models in the subsequent analyses.

### 3.6. Ethical Considerations

The study protocol was approved by the Institutional Review Board of Fooyin University Hospital. In accordance with national regulations and IRB guidelines, the requirement for obtaining informed consent was waived due to the minimal-risk nature of the study and the use of non-identifiable data.

## 4. Results

### 4.1. Participant Characteristics

A total of 355 valid questionnaires were included in the final analysis. The demographic characteristics of the respondents are summarized in Table 1. Most respondents were employed in regional hospitals (38.0%) and district hospitals (35.8%), followed by medical centers (21.4%). More than half of the respondents worked in private hospitals (51.0%), while 36.9% were affiliated with foundation hospitals. The majority of participants were located in southern Taiwan (95.8%). Regarding tenure, 29.3% of respondents had worked in their current organization for 5–10 years, and 16.3% had more than 10 years of experience. Females accounted for 60.0% of the sample, and most respondents were between 20 and 50 years old. The majority held a bachelor’s degree (82.5%).

### 4.2. Descriptive Statistics and Correlation Analysis

Descriptive statistics and Pearson correlation coefficients for the study variables are presented in Table 2. Organizational justice, organizational citizenship behavior, perceived organizational support, and knowledge sharing demonstrated moderate mean values, whereas computer self-efficacy showed relatively higher variability. In addition, diagnostic tests indicated that the assumptions of linear regression, including normality, homoscedasticity, independence of residuals, and absence of multicollinearity, were satisfactorily met.

Correlation analysis revealed that organizational justice was positively correlated with organizational citizenship behavior (r = 0.206, *p* < 0.001) and knowledge sharing (r = 0.295, *p* < 0.001). Perceived organizational support was positively associated with organizational citizenship behavior (r = 0.311, *p* < 0.001) and knowledge sharing (r = 0.382, *p* < 0.001). Organizational citizenship behavior was also positively correlated with knowledge sharing (r = 0.266, *p* < 0.001). In contrast, computer self-efficacy was not significantly correlated with knowledge sharing (r = −0.006, n.s.), although it was positively correlated with organizational citizenship behavior (r = 0.311, *p* < 0.001). These results provide preliminary support for the proposed relationships and indicate no serious multicollinearity concerns among the variables.

### 4.3. Effects of Organizational Justice on Organizational Citizenship Behavior and Knowledge Sharing

Hierarchical regression analyses were conducted to examine the direct and indirect effects of organizational justice on knowledge sharing, with organizational citizenship behavior specified as a mediator. The results are reported in Table 3.

Organizational justice had a significant positive effect on organizational citizenship behavior (β = 0.226, *p* < 0.001), supporting H1. Organizational citizenship behavior also exhibited a significant positive effect on knowledge sharing (β = 0.266, *p* < 0.001), supporting H4. Furthermore, organizational justice was positively associated with knowledge sharing when entered alone into the regression model (β = 0.295, *p* < 0.001). After organizational citizenship behavior was included in the model, the effect of organizational justice on knowledge sharing remained significant but was reduced in magnitude (β = 0.251, *p* < 0.001). These findings indicate that organizational citizenship behavior partially mediates the relationship between organizational justice and knowledge sharing, supporting H5-1.

### 4.4. Effects of Perceived Organizational Support on Organizational Citizenship Behavior and Knowledge Sharing

The mediating role of organizational citizenship behavior in the relationship between perceived organizational support and knowledge sharing was further examined using hierarchical regression analysis. The results are presented in Table 4.

Perceived organizational support was positively associated with organizational citizenship behavior (β = 0.311, *p* < 0.001), supporting H2. Organizational citizenship behavior had a significant positive effect on knowledge sharing (β = 0.266, *p* < 0.001). Perceived organizational support also demonstrated a significant direct effect on knowledge sharing (β = 0.382, *p* < 0.001). When organizational citizenship behavior was added to the regression model, the effect of perceived organizational support on knowledge sharing decreased but remained statistically significant (β = 0.332, *p* < 0.001). These results indicate that organizational citizenship behavior partially mediates the relationship between perceived organizational support and knowledge sharing, thereby supporting H5-2.

### 4.5. Effects of Computer Self-Efficacy on Organizational Citizenship Behavior and Knowledge Sharing

The results of the hierarchical regression analysis examining the role of computer self-efficacy are shown in Table 5. Computer self-efficacy had a significant positive effect on organizational citizenship behavior (β = 0.311, *p* < 0.001), supporting H3. However, computer self-efficacy did not have a significant direct effect on knowledge sharing (β = −0.006, n.s.). When organizational citizenship behavior was included in the regression model, the indirect effect of computer self-efficacy on knowledge sharing through organizational citizenship behavior was not significant. Therefore, organizational citizenship behavior did not mediate the relationship between computer self-efficacy and knowledge sharing, and H5-3 was not supported.

### 4.6. Results of Hypothesis Testing

Overall, the results demonstrate that organizational justice and perceived organizational support influence knowledge sharing both directly and indirectly through organizational citizenship behavior. In contrast, although computer self-efficacy enhances organizational citizenship behavior, it does not contribute to knowledge sharing either directly or indirectly. A summary of hypothesis testing results is provided in Table 6. As summarized in Table 6, H1–H4 were supported, while H5-1 and H5-2 demonstrated partial mediation effects. H5-3 was not supported.

## 5. Discussion and Implications

### 5.1. Discussion of Findings

This study examined the relationships among organizational justice, perceived organizational support, computer self-efficacy, organizational citizenship behavior, and knowledge sharing in healthcare organizations. By positioning organizational citizenship behavior as a mediating mechanism, the findings contribute to the literature by clarifying how organizational conditions and individual capabilities jointly shape discretionary knowledge-sharing behaviors in hospital settings.

The results indicate that organizational justice has a significant positive effect on organizational citizenship behavior, supporting H1. This finding is consistent with a substantial body of prior research demonstrating that fair procedures and respectful treatment encourage employees to engage in extra-role behaviors [13,14,15]. From a social exchange perspective, perceptions of fairness signal organizational trustworthiness and activate reciprocity norms [11], motivating employees to voluntarily contribute beyond formal job requirements. Our findings extend this line of research by confirming that such justice-driven citizenship behaviors remain highly salient in healthcare organizations, where interprofessional collaboration and workload pressures are pervasive.

Similarly, perceived organizational support was found to positively influence organizational citizenship behavior, supporting H2. This result aligns with prior studies suggesting that employees who feel valued and cared for by their organization are more likely to reciprocate through prosocial and cooperative behaviors [12,17]. Recent empirical evidence has further shown that perceived organizational support fosters organizational citizenship behavior and knowledge sharing in contemporary organizational contexts [18]. Our findings corroborate and extend these studies by demonstrating that, in healthcare settings characterized by high emotional labor and stress, organizational support serves as a critical psychological resource that sustains discretionary contributions.

Consistent with prior knowledge management research, organizational citizenship behavior exhibited a significant positive effect on knowledge sharing, supporting H4. This finding echoes earlier studies indicating that individuals who engage in helping and cooperative behaviors are more willing to share knowledge with colleagues [8,9]. More recent research has emphasized that organizational citizenship behavior promotes knowledge sharing by fostering trust-based and relational exchange processes [10]. The present study reinforces this perspective by empirically confirming the central role of citizenship behavior in facilitating voluntary knowledge exchange within healthcare teams.

Beyond reinforcing these findings, recent research further confirms that organizational citizenship behavior promotes knowledge sharing by fostering collaborative norms, psychological safety, and interpersonal trust within organizations [19]. Such evidence aligns with the notion that supportive organizational climates and relational mechanisms help translate discretionary behaviors into active knowledge exchange, strengthening collective competence within healthcare teams.

Regarding individual-level factors, computer self-efficacy was found to positively influence organizational citizenship behavior, supporting H3. This result is consistent with social cognitive theory, which posits that efficacy beliefs shape motivation and proactive behavior [21], as well as prior research linking technology-related self-efficacy to work engagement and helping behaviors [20,25]. However, computer self-efficacy did not have a significant direct or indirect effect on knowledge sharing, and H5-3 was not supported. This finding contrasts with technology-centric perspectives that emphasize individual competence as a primary driver of knowledge exchange and aligns with recent studies suggesting that technological capability alone is insufficient to promote voluntary knowledge sharing [23].

Importantly, the mediation analyses revealed that organizational citizenship behavior partially mediates the relationships between organizational justice and knowledge sharing (H5-1) and between perceived organizational support and knowledge sharing (H5-2). These findings are consistent with prior research highlighting the mediating role of citizenship behavior in translating organizational conditions into knowledge outcomes [13,18]. By empirically demonstrating these pathways in healthcare organizations, this study extends existing knowledge by showing that favorable organizational environments foster knowledge sharing primarily through behavioral mechanisms rather than direct effects alone.

### 5.2. Theoretical Implications

This study contributes to the healthcare management and organizational behavior literature in several ways. First, by integrating organizational justice, perceived organizational support, and computer self-efficacy within a unified framework, this research extends prior studies that have typically examined these antecedents in isolation [2,9]. The findings underscore the primacy of organizational-level factors grounded in social exchange theory over individual technological confidence in shaping discretionary knowledge-sharing behaviors in healthcare contexts. Thus, the contribution of this study lies not in introducing new constructs but in clarifying an integrated behavioral mechanism through which organizational conditions are translated into knowledge sharing in healthcare organizations.

Second, this study advances theoretical understanding by empirically validating organizational citizenship behavior as a key mediating mechanism linking organizational conditions to knowledge sharing. While prior studies have acknowledged the importance of citizenship behaviors [7,16], few have explicitly tested their mediating role in healthcare settings. The results, therefore, refine existing theories by highlighting behavioral pathways through which organizational fairness and support translate into collective knowledge outcomes.

Third, the non-significant mediation effect involving computer self-efficacy contributes to ongoing debates in the knowledge management literature regarding the role of technology-related factors. By demonstrating that self-efficacy does not automatically foster knowledge sharing, this study nuances technology-oriented perspectives and reinforces the importance of relational and contextual factors in healthcare organizations [23].

Beyond confirming established relationships, the present study offers incremental theoretical value by explicating the behavioral mechanism through which organizational conditions are translated into knowledge sharing in healthcare contexts. While prior studies have separately documented the associations between organizational justice, perceived organizational support, organizational citizenship behavior, and knowledge sharing, empirical evidence clarifying how these factors operate together within healthcare organizations remains limited. By positioning organizational citizenship behavior as a mediating mechanism, this study advances existing theory by demonstrating that favorable organizational conditions primarily influence knowledge sharing through discretionary, extra-role behaviors rather than through direct effects alone.

Moreover, although organizational citizenship behavior has often been treated as a multidimensional construct in prior research, this study conceptualized OCB as an overall behavioral orientation to maintain theoretical parsimony and analytical clarity. This approach aligns with the study’s primary objective of identifying a core behavioral pathway rather than differentiating between specific forms of citizenship behavior. Future research may extend this framework by examining more fine-grained dimensions of OCB (e.g., OCB directed toward individuals versus the organization) or by exploring alternative mediators such as tacit knowledge sharing or psychological empowerment, which may further enrich understanding of knowledge exchange dynamics in healthcare settings.

### 5.3. Practical Implications

From a healthcare management perspective, the findings also contribute to a deeper contextual understanding of knowledge sharing by highlighting its relevance to sustainability-oriented outcomes. Knowledge sharing in hospitals encompasses both explicit knowledge (e.g., clinical guidelines, procedural protocols) and tacit knowledge embedded in professional experience and interprofessional interaction. Organizational citizenship behavior facilitates the voluntary exchange of such tacit knowledge, which is often critical for patient safety, service continuity, and adaptive problem solving in complex clinical environments.

By strengthening citizenship behaviors, healthcare organizations may enhance not only internal knowledge flows but also broader organizational outcomes related to care quality, innovation, and system resilience. Accordingly, the present findings suggest that knowledge sharing should be viewed as a behavioral foundation supporting sustainable healthcare management, rather than merely as an information management process.

From a practical standpoint, the findings suggest that healthcare administrators seeking to promote knowledge sharing should prioritize the cultivation of fair and supportive organizational environments. Consistent with prior research, transparent decision-making processes, equitable resource allocation, and respectful leadership practices can strengthen organizational justice perceptions and encourage citizenship behaviors [14,16].

In addition, fostering perceived organizational support—through recognition, emotional support, and responsive management—may be particularly effective in healthcare settings characterized by high job demands [12,18]. While investments in digital infrastructure and training remain important, the findings caution against overreliance on technological solutions alone. Instead, an integrated approach that combines technological competence with trust-building and supportive organizational cultures is more likely to sustain effective knowledge sharing.

The findings further suggest several action-oriented implications for healthcare administrators. First, fairness-enhancing practices, such as transparent decision-making procedures and equitable workload allocation, may foster organizational citizenship behavior by strengthening perceptions of organizational justice. Second, supportive managerial practices, including recognition, emotional support, and responsive leadership, can reinforce perceived organizational support and motivate discretionary contributions among healthcare workers.

Importantly, while digital technologies and knowledge management systems are increasingly adopted in healthcare organizations, the present results indicate that technological competence alone is insufficient to promote knowledge sharing. Instead, the effectiveness of digital knowledge exchange platforms is likely contingent on the presence of supportive organizational climates and citizenship-oriented behaviors. Thus, integrating digital tools with fairness-oriented governance and supportive human resource practices may represent a more sustainable approach to enhancing knowledge sharing in healthcare settings.

### 5.4. Limitations and Future Research Directions

The limitations of this study are consistent with those noted in prior healthcare management research. The cross-sectional design limits causal inference, and future studies may adopt longitudinal or mixed-method approaches to further explore dynamic relationships among organizational conditions, citizenship behavior, and knowledge sharing. Moreover, given the cultural and institutional specificity of the Taiwanese healthcare system, comparative studies across countries would further enrich the literature [5].

Several limitations of this study also point to promising directions for future research. First, the cross-sectional design precludes causal inference; longitudinal or diary-based designs would allow for a more rigorous examination of temporal relationships among organizational conditions, organizational citizenship behavior, and knowledge sharing. Second, future studies may adopt mixed-method approaches by integrating qualitative interviews to capture the contextual and experiential dimensions of knowledge exchange in healthcare settings.

In addition, advanced analytical techniques such as structural equation modeling and multigroup analysis could be employed to test more complex models and to explore potential differences across professional groups or organizational levels. Finally, future research may focus on digital knowledge exchange platforms in hospitals, examining how organizational citizenship behavior facilitates the effective use of technology-enabled knowledge-sharing systems within sustainable healthcare management frameworks.

## 6. Conclusions

This study examined how organizational justice, perceived organizational support, and computer self-efficacy influence knowledge sharing among healthcare workers, with organizational citizenship behavior serving as a mediating mechanism. The findings demonstrate that organizational justice and perceived organizational support promote knowledge sharing both directly and indirectly through organizational citizenship behavior. In contrast, although computer self-efficacy enhances organizational citizenship behavior, it does not significantly contribute to knowledge sharing.

These results underscore the importance of behavioral and relational mechanisms in facilitating voluntary knowledge exchange within healthcare organizations. Knowledge sharing in hospitals is not solely driven by individual technological competence, but is strongly shaped by organizational contexts that foster fairness, support, and discretionary citizenship behaviors. From a theoretical perspective, this study clarifies the behavioral pathway through which organizational conditions are translated into knowledge sharing in healthcare settings.

From a practical standpoint, the findings suggest that healthcare managers should prioritize fairness-enhancing practices and supportive organizational policies to cultivate organizational citizenship behavior and sustain effective knowledge sharing. By fostering a fair and supportive work environment, healthcare organizations may strengthen internal knowledge flows and support long-term organizational learning and sustainability.

## Figures and Tables

**Table 1:** Demographic characteristics of respondents (N = 355).

Variable	Category	*n*	%
Hospital level	Medical center	76	21.4
	Regional hospital	135	38.0
	District hospital	127	35.8
	Others	17	4.8
Ownership	Public	23	6.5
	Foundation	131	36.9
	Private	181	51.0
	Others	20	5.6
Geographic region	Northern Taiwan	7	2.0
	Central Taiwan	6	1.7
	Southern Taiwan	340	95.8
	Eastern Taiwan	2	0.6
Tenure	≤1 year	50	14.1
	1–3 years	83	23.4
	3–5 years	60	16.9
	5–10 years	104	29.3
	≥10 years	58	16.3
Gender	Male	142	40.0
	Female	213	60.0
Age	20–30 years	125	35.2
	31–40 years	89	25.1
	41–50 years	96	27.0
	51–60 years	40	11.3
	≥60 years	5	1.4
Education level	Junior college	32	9.0
	Bachelor’s degree	293	82.5
	Master’s degree	27	7.6
	Doctoral degree	3	0.8

**Table 2:** Descriptive statistics and correlations among study variables.

Variable	Mean	SD	1	2	3	4	5
1. Organizational Justice	3.461	0.624	1				
2. Organizational Citizenship Behavior	3.834	0.414	0.206 ***	1			
3. Knowledge Sharing	3.733	0.493	0.295 ***	0.266 ***	1		
4. Perceived Organizational Support	3.498	0.645	0.459 ***	0.311 ***	0.382 ***	1	
5. Computer Self-Efficacy	3.756	0.758	0.033	0.311 ***	−0.006	0.019	1

Note: *** *p* < 0.001.

**Table 3:** Hierarchical regression analysis predicting knowledge sharing (organizational justice as predictor).

Variables	Model 1 (OCB)	Model 2 (KS)	Model 3 (KS)	Model 4 (KS)
Organizational Justice	0.226 ***	–	0.295 ***	0.251 ***
Organizational Citizenship Behavior	–	0.266 ***	–	0.214 ***
F-value	18.976 ***	26.784 ***	33.567 ***	26.453 ***
R^2^	0.051	0.071	0.087	0.131
Adjusted R^2^	0.048	0.068	0.084	0.126

Note: OCB = organizational citizenship behavior; KS = knowledge sharing. *** *p* < 0.001. Reported coefficients are standardized beta values.

**Table 4:** Hierarchical regression analysis predicting knowledge sharing (perceived organizational support as predictor).

Variables	Model 1 (OCB)	Model 2 (KS)	Model 3 (KS)	Model 4 (KS)
Perceived Organizational Support	0.311 ***	–	0.382 ***	0.332 ***
Organizational Citizenship Behavior	–	0.266 ***	–	0.162 ***
F-value	37.695 ***	26.784 ***	60.446 ***	36.061 ***
R^2^	0.096	0.071	0.146	0.170
Adjusted R^2^	0.094	0.068	0.144	0.165

Note: *** *p* < 0.001. Reported coefficients are standardized beta values.

**Table 5:** Hierarchical regression analysis predicting knowledge sharing (computer self-efficacy as predictor).

Variables	Model 1 (OCB)	Model 2 (KS)	Model 3 (KS)	Model 4 (KS)
Computer Self-Efficacy	0.311 ***	–	−0.006	−0.098
Organizational Citizenship Behavior	–	0.266 ***	–	0.298 ***
F-value	37.701 ***	26.784 ***	0.012	15.134 ***
R^2^	0.096	0.071	0.000	0.079
Adjusted R^2^	0.094	0.068	−0.003	0.074

Note: *** *p* < 0.001. Reported coefficients are standardized beta values.

**Table 6:** Summary of hypothesis testing.

Hypothesis	Proposed Relationship	Result
H1	Organizational justice → Organizational citizenship behavior	Supported
H2	Perceived organizational support → Organizational citizenship behavior	Supported
H3	Computer self-efficacy → Organizational citizenship behavior	Supported
H4	Organizational citizenship behavior → Knowledge sharing	Supported
H5-1	Organizational citizenship behavior mediates the relationship between organizational justice and knowledge sharing	Supported
H5-2	Organizational citizenship behavior mediates the relationship between perceived organizational support and knowledge sharing	Supported
H5-3	Organizational citizenship behavior mediates the relationship between computer self-efficacy and knowledge sharing	Not supported

Note: H1–H4 were tested using hierarchical regression analysis. Mediation effects (H5-1–H5-3) were examined using hierarchical regression analysis with a bootstrapping approach. “Supported” indicates that the proposed relationship or indirect effect was statistically significant at conventional levels.

## Data Availability

The data presented in this study are not publicly available due to privacy and confidentiality considerations. However, the datasets are available from the corresponding author upon reasonable request.
